# Node deployment optimization of underwater wireless sensor networks using intelligent optimization algorithm and robot collaboration

**DOI:** 10.1038/s41598-023-43272-x

**Published:** 2023-09-23

**Authors:** Yangmei Zhang, Zhouzhou Liu, Yang Bi

**Affiliations:** 1 School of Electronic Engineering, Xi’an Aeronautical Institute, Xi’an, 710077 China; 2 School of Computer Science, Xi’an Aeronautical Institute, Xi’an, 710077 China; 3 Department of Science and Technology, Xi’an Aeronautical Institute, Xi’an, 710077 China

**Keywords:** Energy science and technology, Engineering, Materials science, Mathematics and computing, Nanoscience and technology, Optics and photonics, Physics

## Abstract

This study aims to optimize the node deployment of underwater wireless sensor networks (UWSNs) using intelligent optimization algorithms and robot collaboration technology to enhance network performance and coverage. The study employs the chemical reaction optimization (CRO) algorithm, which combines the advantages of genetic algorithms, simulated annealing algorithms, and ant colony algorithms. The CRO algorithm is enhanced through a structure correction function to determine the optimal node deployment scheme to achieve effective and optimal coverage control of the UWSN. Additionally, the flexibility and autonomy of robots are leveraged to improve the efficiency of node deployment and address the unique challenges posed by the underwater environment. Furthermore, the study conducts a comparative analysis of different intelligent optimization algorithms and demonstrates the effectiveness and advantages of the enhanced CRO algorithm in optimizing node deployment for UWSNs. The study findings reveal that the improved algorithm achieves an average coverage rate of 95.66%, significantly outperforming traditional intelligent optimization algorithms. The coverage of UWSNs can be significantly improved by utilizing the enhanced CRO algorithm and robot collaboration technology for node deployment optimization, which offers an effective approach for achieving optimal node deployment. Moreover, the rational deployment of nodes enhances the monitoring capability, resource utilization efficiency, and accuracy of environmental monitoring in underwater networks. The results of this study hold great practical significance for underwater environment monitoring, marine resource exploration, and marine scientific research.

## Introduction

In recent years, rapid advancements in science and technology have presented promising opportunities for the integration of intelligent optimization algorithms and robot collaboration technology. Intelligent optimization algorithms are computational techniques that simulate natural organisms and group behavior to solve complex problems. Examples of such algorithms include genetic algorithms^1^, particle swarm optimization^2^, and ant colony optimization^3^. These algorithms have demonstrated remarkable achievements in addressing combinatorial optimization problems, scheduling problems, and path planning. On the other hand, robot collaboration technology refers to the collaborative problem-solving capabilities of multi-robot systems. This technology enhances task efficiency and flexibility, and its applications span various domains, such as flexible manufacturing systems, logistics distribution, and environmental monitoring. Researchers are actively exploring the combination of intelligent optimization algorithms and robot collaboration technology, aiming to achieve improved system performance in diverse fields, including ocean exploration and smart cities^4,5^.

Underwater wireless sensor networks (UWSNs) have emerged as a vital technology in the fields of ocean observation, underwater communication, and environmental monitoring^6^. However, the complex underwater environment and the limited energy and communication range of sensor nodes pose challenges to optimizing node deployment and ensuring network performance. Researchers have proposed various solutions to address this issue, including node deployment methods based on strategies such as direction optimization, energy balance, and adaptive mobility. The primary objectives of current research efforts are to achieve efficient energy utilization, optimize network coverage, and enhance communication quality to cater to diverse application scenarios, such as ocean observation and underwater detection. Therefore, it is crucial to continue exploring innovative methods for optimizing the deployment of nodes in UWSNs, as it holds significant implications for the advancement of these networks and their practical applications.

The study integrates the intelligent optimization algorithm and the collaborative robot strategy to enhance the efficiency of node deployment in UWSNs. Specifically, the study leverages the chemical reaction optimization (CRO) algorithm with a focus on optimizing the structure modification function. Additionally, the flexibility and autonomy of robots are utilized to achieve more efficient node deployment. The primary logical framework of the study is outlined as follows. “Introduction” section of the study acquaints the reader with the backdrop of intelligent optimization algorithms and strategies for robot collaboration. “Related works” section concisely reviews the most recent literature pertaining to wireless sensor networks and intelligent optimization algorithms. “Design and optimization of wireless sensor node deployment scheme based on intelligent optimization algorithm” section delves into an in-depth examination of the communication efficiency within the sensor node network, achieved through the formulation of a robot collaboration-oriented node deployment strategy. Subsequently, “Results and discussion” section undertakes a comprehensive comparative analysis of the optimization efficacy across diverse sensor nodes. Finally, “Conclusion” section encapsulates the research findings, culminating in a methodical synthesis of the conclusions drawn from the study. The findings of this study hold practical significance in enhancing the data transmission performance of UWSNs, providing valuable insights for further advancements in this field.

## Related works

### Recent literature of UWSNs and intelligent optimization algorithms

The increasing application of UWSNs (UWSNs) in ocean monitoring, resource exploration, and military domains has brought about extensive development in the field. However, constructing and managing underwater networks present challenges due to the complex underwater environment and limited transmission capabilities. Jawad et al.^7^ conducted a review on the implementation of energy-efficient wireless sensor networks within the context of precision agriculture. Their discourse primarily centered around strategies aimed at optimizing energy consumption for monitoring and regulating the agricultural milieu through wireless sensor networks. They thoroughly examined prevailing technologies and remedies, encompassing energy-efficient algorithms, methodologies for energy harvesting and conversion, and more. In a similar vein, Kobo et al.^8^ embarked on a survey that delved into the realm of software-defined wireless sensor networks, dissecting both the challenges and design imperatives in this domain. Their work entailed an evaluation of the application of software-defined networking within wireless sensor networks, encompassing domains such as network administration, security considerations, and the quest for energy efficiency. Lilhore et al.^9^ proposed a low-energy routing protocol with deep control. This protocol monitors the depth of underwater sensor nodes in real-time to regulate the number of nodes, depth spanning, and movement speed, thereby enabling precise control of routing and effective energy management. The research findings reveal that this protocol significantly reduces node energy consumption while enhancing network efficiency compared to traditional routing protocols. Subramani et al.^10^ proposed a meta-heuristic clustering routing protocol. By leveraging meta-heuristic algorithms to optimize UWSNs, they introduce a new and more efficient routing protocol that achieves a balance between energy consumption and network efficiency. The research results indicated that this protocol significantly improves the real-time performance of data transmission and overall network performance compared to traditional routing protocols.

With the rapid advancement of artificial intelligence technology, optimization algorithms have emerged as crucial tools for solving complex problems. These algorithms draw inspiration from natural principles and behaviors to achieve efficient global optimization and have demonstrated significant success in diverse application domains. For instance, Zhang et al.^11^ proposed a real-time and pervasive network attack detection method based on deep belief networks and support vector machines. This method employs a deep belief network to extract data features and combines support vector machines for classification and judgment, enabling real-time network data monitoring and attack detection. Experimental results showcase the method’s commendable performance in terms of both detection accuracy and processing speed, effectively enhancing network security. In another study, Kumar et al.^12^ utilized the ant colony optimization algorithm and the Internet of Things (IoT) technology to design an intelligent traffic control system. By integrating the IoT technology at intersections and optimizing the control system using the ant colony optimization algorithm, they achieved significant improvements in the efficiency of intelligent traffic control. Furthermore, Khishe et al.^13^ introduced the orangutan optimization algorithm, which simulates the behavior and social structure of orangutans to achieve efficient global optimization. By employing regional division and detailed analysis to mimic population behavior, this algorithm demonstrated high efficiency in tackling complex problems. Rana et al.^14^ comprehensively reviewed the whale optimization algorithm and its applications in various fields. This algorithm offers superior solutions by integrating the relevant behavior of whales into the search process. Research has shown its successful implementation across multiple domains. Pereira et al.^15^ employed the Lichtenberg optimization algorithm to identify crack tip locations in thin plate-like structures. The author analyzed the transmission characteristics of thin plates and designed the Lichtenberg optimization algorithm to locate crack tips accurately. Maheshwari et al.^16^ combined the butterfly optimization algorithm and the ant colony algorithm to propose an energy-efficient cluster routing protocol for wireless sensor networks. The butterfly algorithm selects the data head node, while the ant colony algorithm determines the route, resulting in improved energy efficiency and data transmission efficiency within the network. Finally, Vinitha et al.^17^ developed a secure and energy-saving multi-hop routing protocol. This protocol utilizes an optimization algorithm based on the Taylor function for path selection, considering factors such as the remaining power of sensor nodes and the signal-to-noise ratio. The research findings highlight the algorithm’s ability to significantly enhance the energy efficiency performance of sensor networks.

In summary, the landscape of wireless sensor networks has witnessed a proliferation of diverse optimization algorithms that aim to address intricate challenges. Inspired by natural principles and behaviors, these algorithms have exhibited substantial efficacy in accomplishing global optimization tasks. Concurrently, the rapid evolution of artificial intelligence technology has propelled optimization algorithms into a pivotal role in tackling complex conundrums. By harnessing the capabilities of intelligent optimization algorithms in conjunction with robotic collaboration, this research stands poised to capitalize on existing technological reservoirs and solutions. The objective is to attain optimal configurations for node deployments, thereby engendering enhancements in network performance, energy efficiency, and real-time operability. The resultant insights and direction will extend pivotal guidance for subsequent research and underpin pragmatic applications within similar domains.

### Recent studies related to sensor network node deployment strategies

As wireless sensor networks serve as crucial platforms for information collection and transmission in various domains such as smart cities, agriculture, and environmental monitoring, the accuracy and rationality of node deployment play pivotal roles in determining network performance and application effectiveness. Consequently, studying and optimizing node deployment in wireless sensor networks hold significant practical significance. Bala et al.^18^ conducted a comprehensive review of challenges and issues encountered in wireless sensor networks. They explored concerns related to sensor network architecture, node communication, and energy management, offering potential solutions to address these challenges. The findings highlight the wide range of applications for wireless sensor networks, emphasizing the importance of optimizing node deployment and energy management to ensure network stability and performance. Liu et al.^19^ introduced an ant colony optimization algorithm incorporating a greedy migration mechanism for wireless sensor network node deployment. They elucidated the algorithm’s principles, its practical realization, and subsequent performance evaluation. The outcomes underscored the algorithm’s efficacy in heightening both coverage and energy efficiency within sensor networks. In a complementary vein, Abdollahzadeh et al.^20^ conducted a comprehensive survey of deployment strategies within wireless sensor networks. They meticulously distilled a gamut of deployment techniques while meticulously assessing their respective merits and demerits. Additionally, the article delves into the nuanced influence of diverse application scenarios and network topologies on strategic deployment approaches. Sharma et al.^21^ reviewed the application of machine learning in wireless sensor networks for smart cities. The paper discussed the utilization of machine learning techniques in data processing, signal processing, and node deployment, summarizing the current research progress in this field. The results underscore the capability of machine learning techniques to enhance the adaptability and performance of wireless sensor networks in smart city applications. Li et al.^22^ proposed a node deployment algorithm based on data fusion and evidence theory. By integrating node data and employing evidence theory to evaluate node weights, the algorithm improves the accuracy and reliability of deployment. The findings demonstrate that the algorithm enables more precise and reliable deployment of sensor nodes. Mohar et al.^23^ focused on optimal sensor node deployment. They proposed an optimal deployment scheme utilizing the bat algorithm for optimization. The results indicate that the scheme minimizes the number of sensor nodes while ensuring node coverage, thereby enhancing energy efficiency and performance in wireless sensor networks.

To encapsulate, within wireless sensor networks, optimizing node deployment emerges as a pivotal approach to engender network stability, augment performance, and enhance application outcomes. This pertinence resonates notably within UWSNs, where the utilization of intelligent optimization algorithms coupled with robotic collaboration stands as a central thrust of the ongoing investigation. The present research paradigm is geared towards the automated orchestration and optimization of node placements through the agency of intelligent optimization algorithms synergistically harnessed with robotic collaboration. This concerted approach aims to engender resourceful node deployment, thereby amplifying network coverage and efficacy. Additionally, the integration of robotic collaboration expedites and enhances the precision of node deployment, circumventing laborious and imprecise manual interventions while bolstering the efficiency and dependability of the deployment procedure. In essence, the exploration of the amalgamation of intelligent optimization algorithms and robotic collaboration in UWSN node deployment bears significant theoretical and pragmatic import.

## Design and optimization of wireless sensor node deployment scheme based on intelligent optimization algorithm

### Intelligent optimization algorithms and machine collaboration for UWSNs node deployment scheme

**Figure 1:**
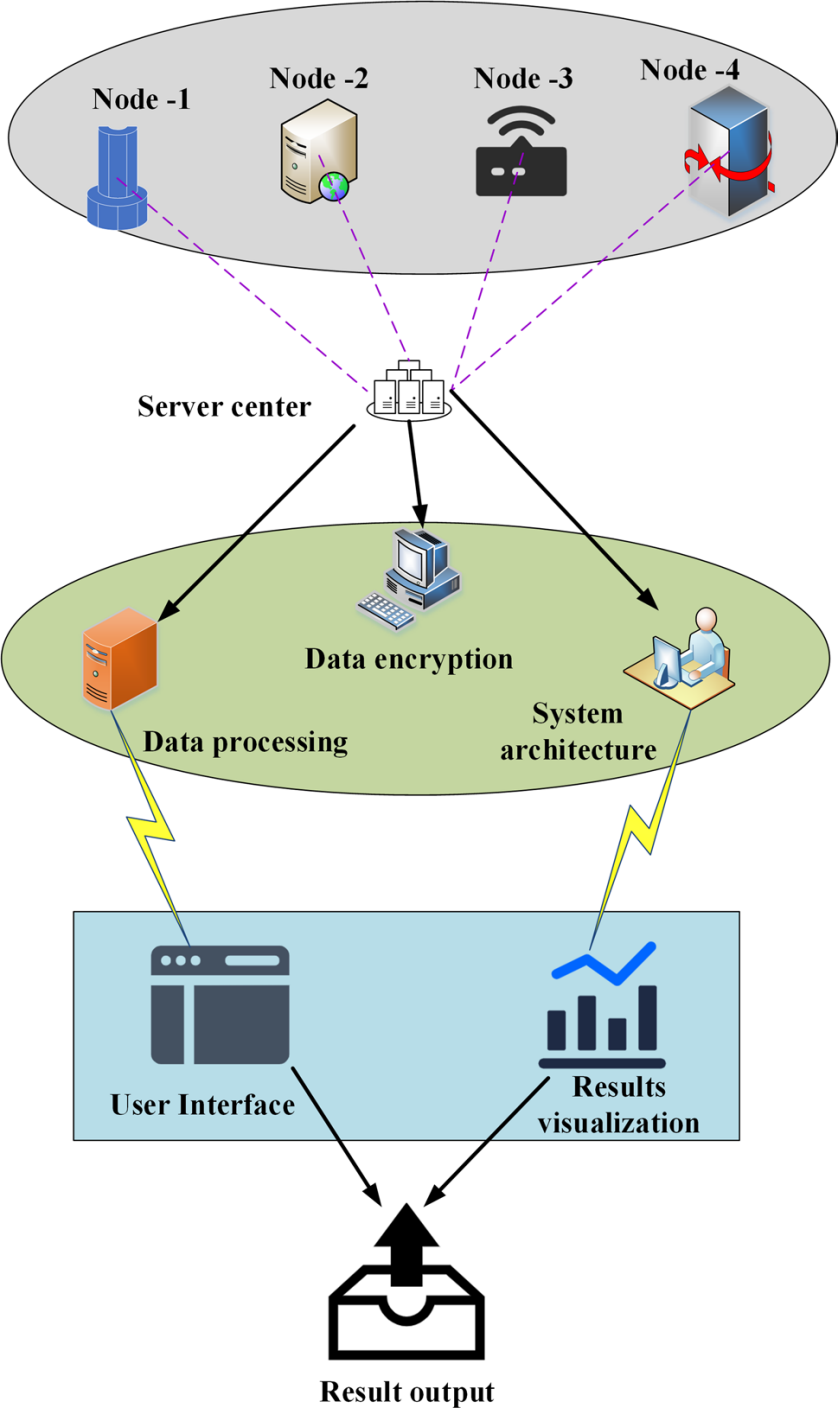
System structure of node deployment of UWSNs.

In the research focused on optimizing the node deployment of UWSNs using intelligent optimization algorithms and robot collaboration, the performance of UWSNs is enhanced through the utilization of multimedia information perception and acquisition technology^24,25^. This process involves the deployment of wireless sensor nodes to gather various seabed environmental information, including data obtained from scalar sensors and image sensors. The optimal deployment locations for sensor nodes can be efficiently determined by employing an intelligent optimization algorithm, leading to the optimization of network coverage, energy consumption, and communication quality. Figure 1 illustrates the overall structure of the sensor network’s node deployment, facilitating the analysis of the intelligent optimization algorithm and the network’s structure.

Figure 1 depicts the spatial arrangement and dispersion of multiple nodes situated underwater. These nodes are strategically positioned at various underwater locales, interlinked through the utilization of wireless communication technology to facilitate seamless data transmission. Furthermore, the distinct sensor node variants are interlinked primarily via a central controller or a base station. Notably, communication nodes play a pivotal role in orchestrating and overseeing the entirety of the UWSN.

### Innovative design and investigation of node arrangement scheme of underwater sensing network

**Figure 2:**
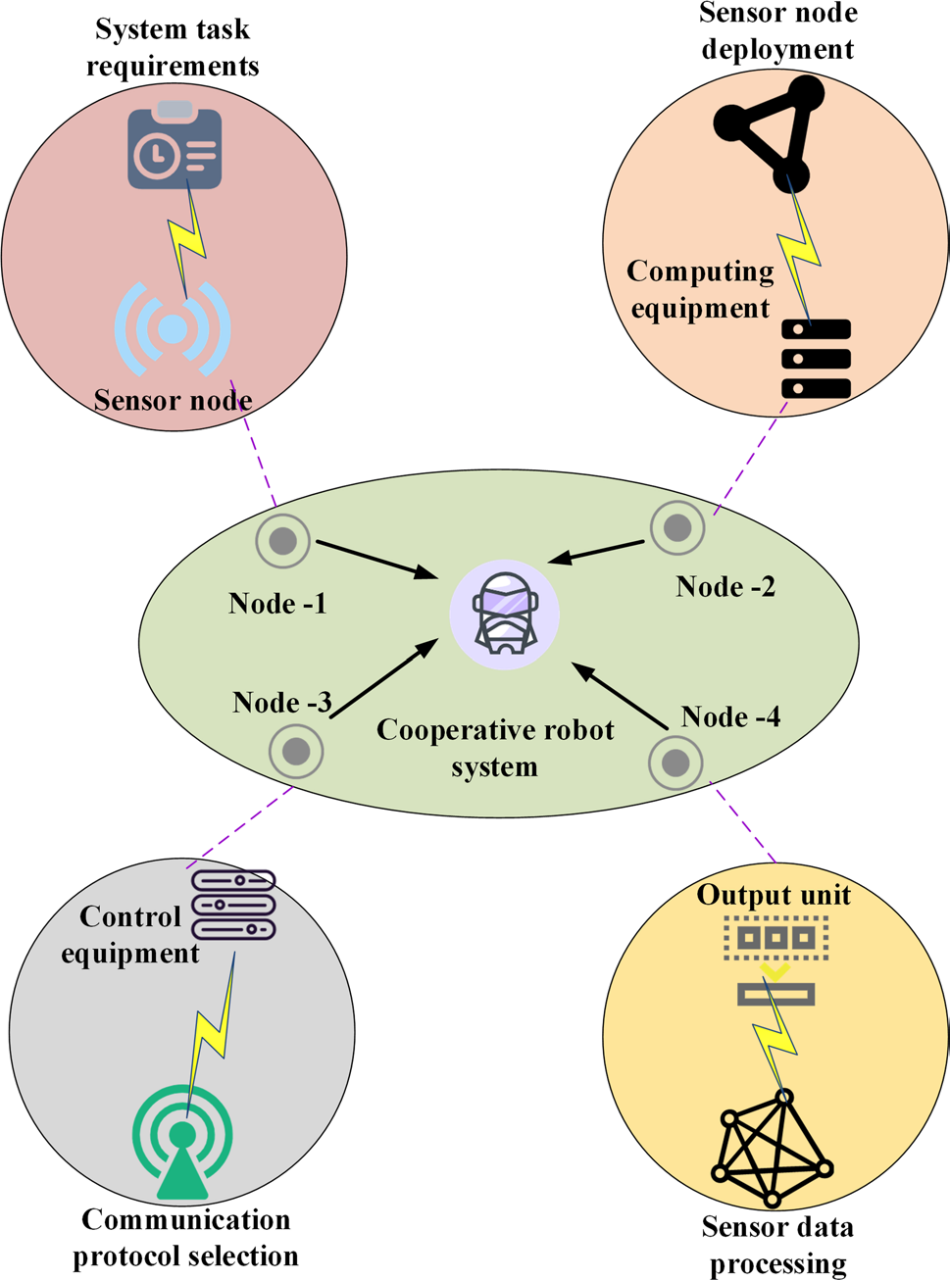
Optimal design scheme of node deployment for robot collaboration.

This study introduces an innovative solution for addressing the node deployment problem in UWSNs through the integration of intelligent optimization algorithms and robot collaboration. An adaptive enhanced hybrid intelligent optimization algorithm is proposed to account for the specific challenges posed by the underwater environment. This algorithm combines the genetic and particle swarm algorithms to generate an initial layout that considers important indicators such as network coverage, communication quality, and energy consumption^26–28^. Appropriate coordinates are derived for each sensor node during the initialization phase based on the preliminary planning outcomes. An autonomous or semi-autonomous underwater robot system with multi-manipulator cooperative operation is designed to achieve precise node deployment. The system employs a disseminated coordination strategy to dynamically control the spacing between robots, ensuring a rapid and stable deployment process. Additionally, a distributed fault detection mechanism is established to enhance inter-node communication reliability and address potential node failures. In situations where coverage gaps occur, preset backup nodes or the adjustment of adjacent node positions are utilized to fill these gaps. The optimized design structure of the node deployment scheme, specifically tailored for robot collaboration, is depicted in Fig. 2.

Figure 2 illustrates an optimized scheme for node arrangement, facilitating effective robot collaboration. These nodes can encompass robots, sensors, or other pertinent devices. This arrangement scheme can be optimized to attain optimal robot collaboration by depicting the spatial disposition and distribution of nodes. Additionally, nodes can engage in information exchange and collaborative endeavors through wireless communication, thereby enhancing collaboration efficiency, curtailing communication latency, and bolstering system resilience.

### Communication performance analysis and optimization design for UWSN node deployment

**Figure 3:**
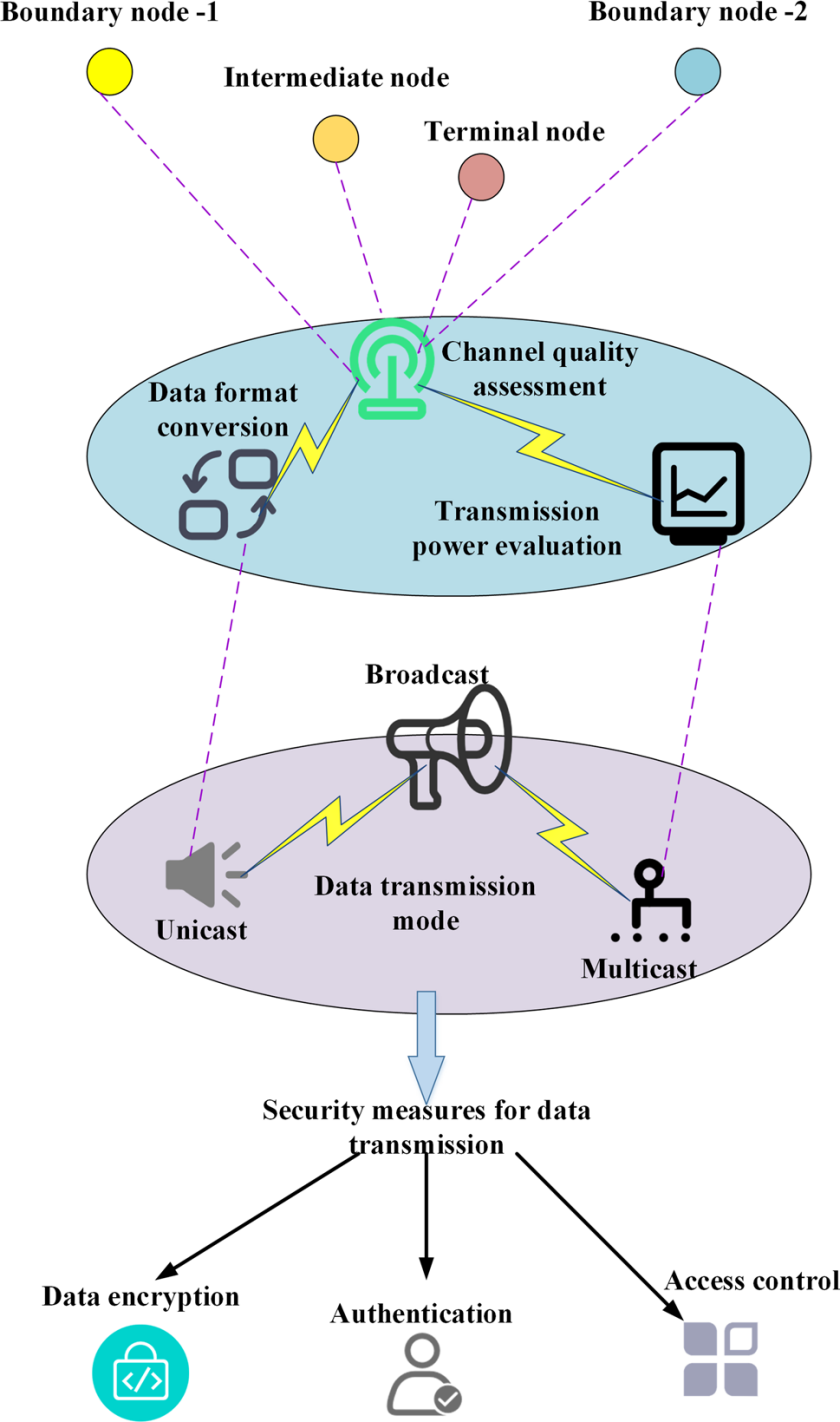
Communication system structure of sensor node networks.

During the deployment of sensor network nodes, it is crucial to assess the overall communication performance of the network. An underwater acoustic communication protocol based on multi-hop routing and adaptive mode selection is employed to achieve optimal communication effectiveness. This protocol allows for the adjustment of parameters such as transmission power, coding method, and beam pointing. Moreover, a link quality assessment method based on received signal strength indication is designed, considering factors such as spatial correlation, time correlation, and frequency correlation. This method enables dynamic routing strategy adjustments to ensure efficient communication^29,30^. A cooperation mechanism-based energy-saving strategy is investigated to conserve energy. This strategy leverages high-performance nodes to assist low-performance nodes in data processing and communication tasks, thus balancing the network load and extending the network’s lifespan. A security mechanism is also introduced, which involves key distribution and encryption authentication. This mechanism safeguards the network against malicious attacks or eavesdropping, ensuring secure data transmission. The overall communication system structure of the sensor node network is depicted in Fig. 3, providing an overview of the communication components and their interconnections.

As depicted in Fig. 3, the sensor node network comprises numerous sensor nodes dedicated to gathering and relaying environmental data. Wireless communication facilitates connectivity among these sensor nodes. Diverse types of sensor nodes are assigned the task of sensing and gathering distinct environmental data. A central controller assumes the role of managing and orchestrating communication and data transmission among the sensor nodes.

### Optimization method and simulation evaluation of UWSN node deployment based on intelligent optimization algorithm and robot collaboration

**Figure 4:**
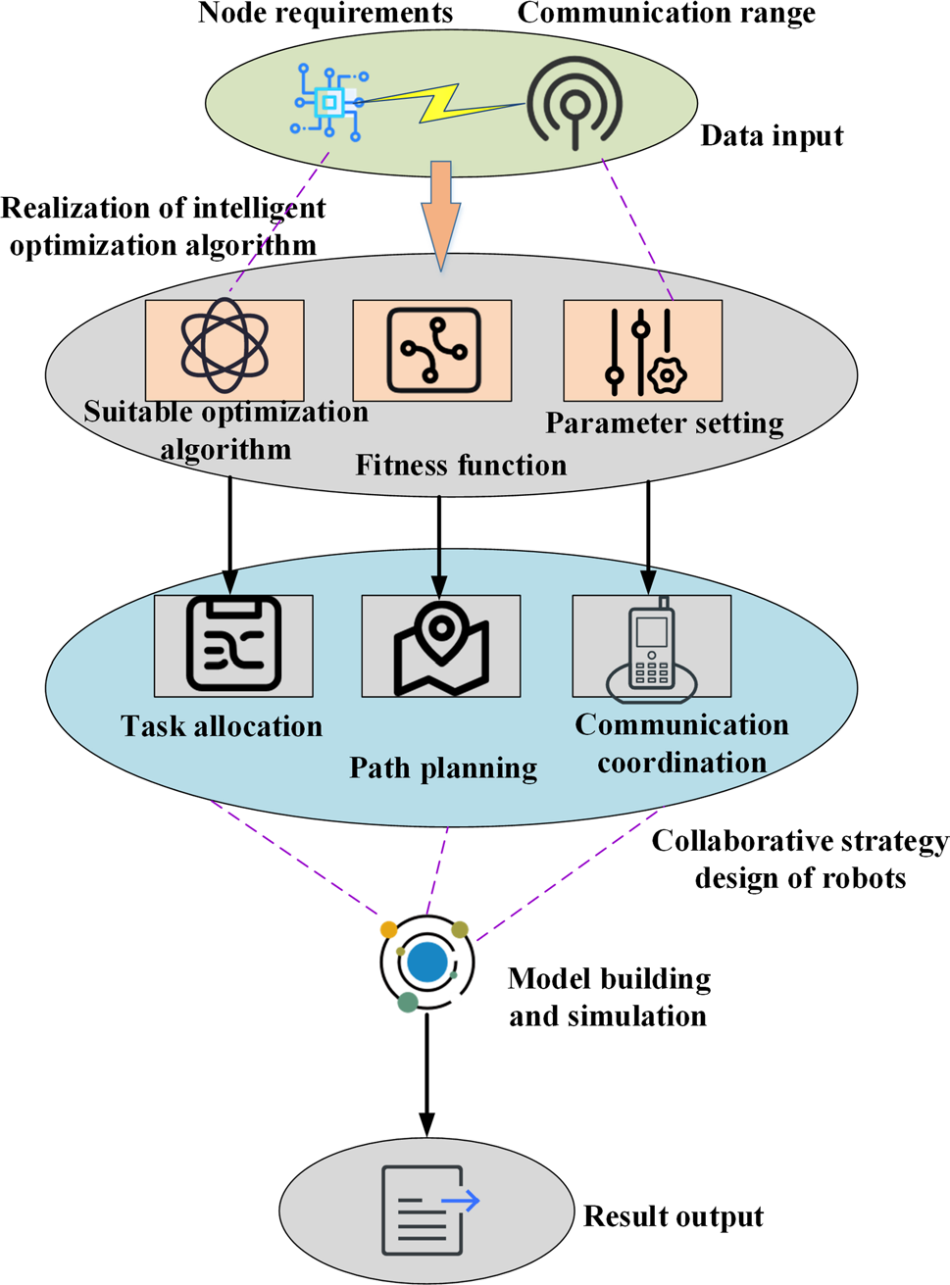
The overall steps and optimization process of sensor network node deployment.

This study presents a node deployment optimization method for UWSNs, utilizing intelligent optimization algorithms and robot collaboration. The experimental simulation design involves the establishment of an appropriate environment and model. Factors such as underwater temperature, salinity, flow velocity, as well as sensor node characteristics, including energy consumption, transmission power, failure probability, and communication range, are systematically analyzed to determine the network scale, simulation area size, and communication distance, serving as simulation parameters. An intelligent optimization algorithm is employed to optimize the deployment of nodes in the UWSN. A collaborative strategy with multiple robots is also introduced to facilitate dynamic node adjustment and maintenance. A comparative analysis of various optimization algorithms is conducted throughout the simulation process, focusing on node deployment optimization. The feasibility and effectiveness of collaborative robot operation are also evaluated in alignment with real-world scenarios. Figure 4 illustrates the overall steps and optimization process involved in network node deployment, providing a comprehensive overview of the algorithm optimization and design procedure.

Figure 4 presents a comprehensive, sequential depiction of the sensor network node deployment process. This inclusive portrayal encompasses specific procedural stages, such as planning, node selection, and spatial arrangement. By factoring in multiple considerations, including energy consumption, communication range, and node coverage, the deployment of sensor network nodes aims to identify the most optimal deployment scheme. The overarching structure of sensor network node deployment is achieved and refined through the integration of task allocation, path planning, and task coordination mechanisms.

Furthermore, this study investigates the performance of the proposed intelligent machine cooperative node (IMCN) deployment model based on the CRO algorithm. A comparative analysis is conducted between the IMCN model and other node deployment models, including the genetic algorithm node (GAN), ant colony algorithm node (ACAN), particle swarm algorithm node (PSAN), convolutional neural network node (CNNN), and simulated annealing algorithm node (SAAN). In accordance with the pertinent reference^31^, the IMCN setup process involves the determination of several key parameters. Specifically, the configuration encompasses establishing 10 nodes, allocating 3 underwater network layers, and specifying a simulation area measuring 500 m × 500 m. Each node’s energy consumption is calibrated at 1.5 J/s, while their transmission power is set at 10 dBm, aligning with communication range stipulations and transmission capabilities. Furthermore, the communication range of each node is calibrated at 100 m, as dictated by the node’s communication range requirement and corroborated by experimental data. The comparison considers various aspects, such as network energy consumption, system deployment time, target area coverage, data transmission success rate, model failure interruption probability, and network throughput. The study aims to identify their respective strengths and weaknesses by evaluating the performance of different node deployment models in the sensor network. This analysis provides valuable insights for practical applications, enabling informed decisions on selecting the most suitable node deployment model (Supplementary file).

## Results and discussion

### Performance evaluation and comparison of node deployment optimization algorithms for UWSNs based on energy consumption

**Figure 5:**
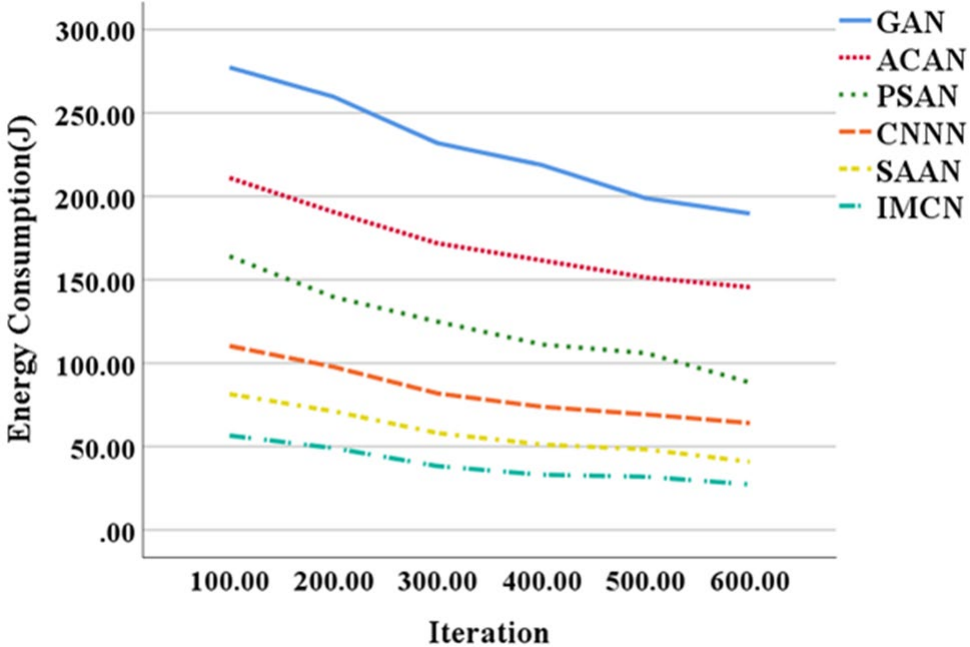
Trends of node deployment energy consumption data of UWSNs constructed by different optimization algorithms.

**Figure 6:**
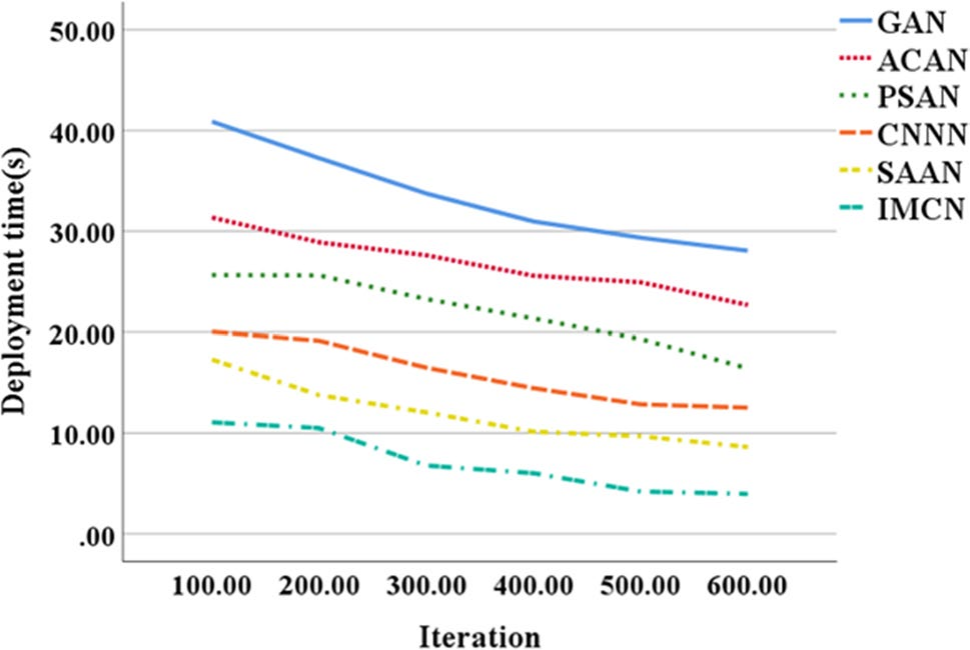
Changing trend of node deployment time of UWSN constructed by different optimization algorithms.

Figure 5 illustrates the energy consumption data trends for node deployment in UWSNs using various optimization algorithms, while Fig. 6 depicts the corresponding trends in node deployment time. These visual representations enable a comprehensive comparison of the performance among different optimization algorithms, facilitating the evaluation and selection of the most appropriate algorithm for specific application scenarios.

Figure 5 shows that the energy consumption values for node deployment using all algorithms exhibit a consistent downward trend. This trend indicates that as the number of iterations increases, the performance of the algorithms improves. At 600 iterations, the energy consumption for node deployment based on GAN is 189 J; for ACAN, it is 145 J; for IMCN, it is 27 J. Based on these data and analysis, it can be concluded that the IMCN algorithm, which is based on the CRO algorithm, outperforms the other algorithms in terms of node deployment energy consumption. It demonstrates faster convergence speed and lower energy consumption, making it an effective choice for algorithm selection. The IMCN algorithm demonstrates superior performance metrics in comparison to alternative algorithms, leading to substantial reductions in energy consumption and notable enhancements in network efficiency. This characteristic holds particular significance in resource-constrained environments like IoT and sensor networks. Consequently, the IMCN algorithm emerges as a favorable and recommended choice for the selection of node deployment algorithms.

As depicted in Fig. 6, the node deployment time for UWSNs constructed using different optimization algorithms gradually decreases with an increasing number of model iterations. At 100 iterations, the network nodes deployed based on IMCN require 11 s for deployment. However, with 600 iterations, the node deployment time for the IMCN-based UWSN reduces to a mere 4 s. This observation leads to the conclusion that, with an equal number of iterations, the IMCN model based on the CRO algorithm exhibits superior performance in terms of node deployment time in UWSNs. This improvement in deployment time contributes to enhanced efficiency and reliability in UWSNs, opening up new possibilities for future applications in areas such as deep-sea exploration. The deployment time of nodes, as generated by distinct algorithms, exhibits temporal variations. The depicted figure serves as a comparative assessment of diverse optimization algorithms with regard to the duration of node deployment. Through this evaluation of node deployment time across different algorithms, their respective efficiency and speed can be ascertained. The discernible verification of the IMCN model’s superiority is evident upon comparison with several other models.

### Performance evaluation and comparison of UWSN deployment optimization algorithms based on network coverage and data transmission

**Figure 7:**
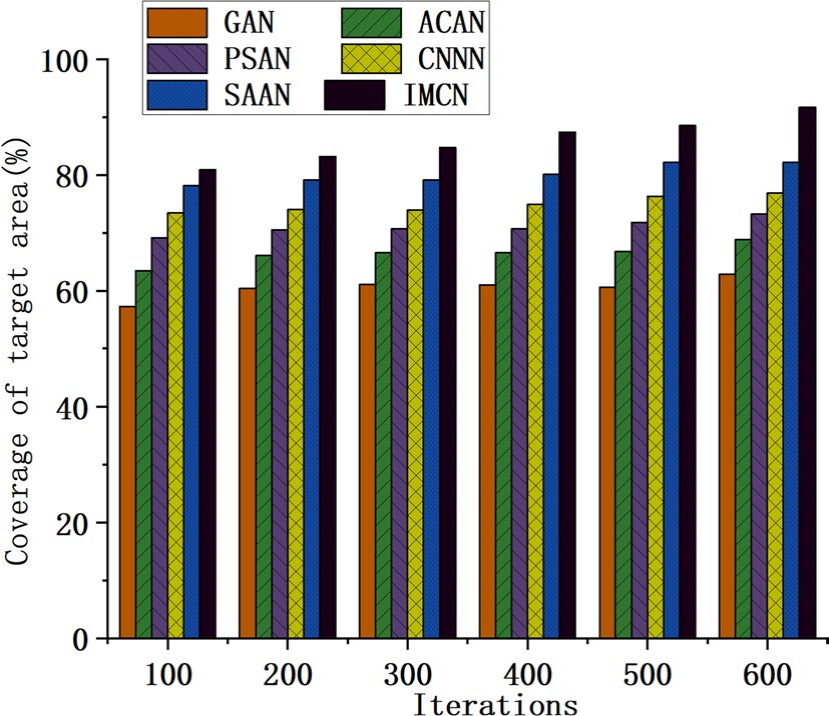
Variation trend of the coverage data of the UWSN to the target area constructed by different optimization algorithms.

**Figure 8:**
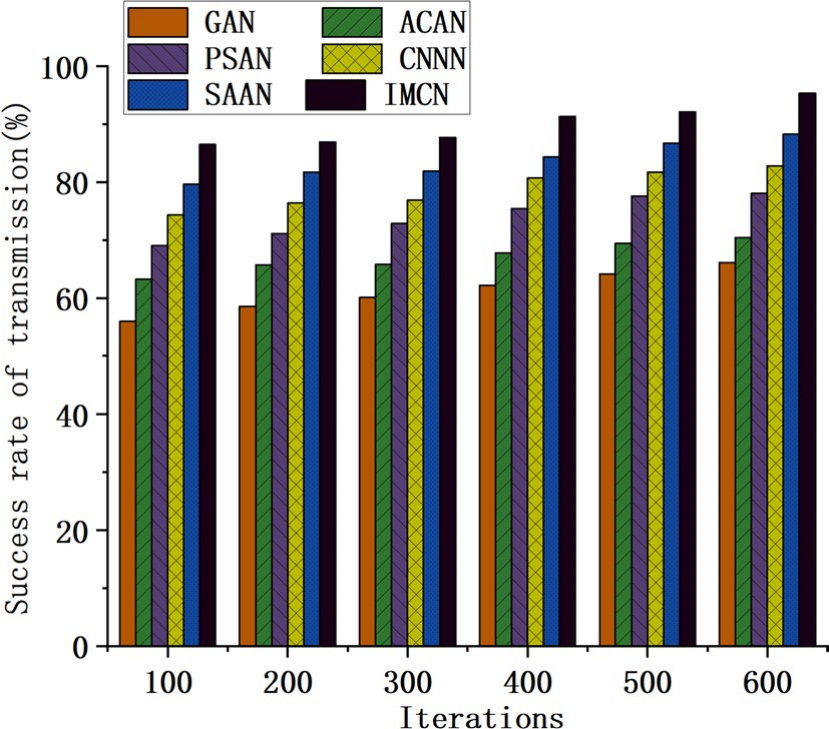
Variation trend of the data transmission success rate of the UWSN constructed by different optimization algorithms.

In the study of UWSNs, accurate node deployment and selection of optimization algorithms are vital for system performance. This study introduces two performance indicators: sensor network coverage and data transmission success rate to assess the impact of various algorithms in different aspects. The sensor network deployment performance of different optimization algorithms is analyzed accordingly. Figure 7 presents the trend of coverage data for the target area when employing different optimization algorithms to construct the UWSN. Conversely, Fig. 8 illustrates the corresponding trend of data transmission success rate. These figures provide visual representations that aid in evaluating the performance of different optimization algorithms, facilitating the selection of the most suitable algorithm for specific application scenarios.

As depicted in Fig. 7, the coverage of UWSNs constructed using different optimization algorithms steadily improves with an increase in the number of iterations. Notably, the IMCN-based network exhibits the highest coverage, reaching 80.91% at 100 iterations and 91.71% at 600 iterations. While SAAN and PSAN initially perform well at lower iteration counts, their performance is surpassed by GAN, ACAN, and IMCN as the number of iterations increases. The study findings demonstrate that the improved CRO algorithm achieves an average coverage rate of 95.66%, significantly higher than other traditional intelligent optimization algorithms. These results highlight the effectiveness of the IMCN algorithm based on CRO in enhancing the coverage of UWSNs. Upon further analysis of these findings, a noteworthy disparity becomes apparent in the coverage achieved by distinct optimization algorithms for constructing UWSNs. While SAAN and PSAN exhibit competitiveness in the initial phase, their performance progressively lags behind other algorithms. Notably, the networks established through the utilization of GAN, ACAN, and IMCN algorithms exhibit steadily enhanced coverage as the number of iterations increases.

As depicted in Fig. 8, the data transmission success rate of network models constructed using different optimization algorithms exhibits an increasing trend with increased model iterations. Notably, when the number of iterations reaches 600, the success rate of data transmission based on GAN is 66.13%, ACAN achieves a success rate of 70.44%, while IMCN attains an impressive success rate of 95.35%. Thus, it can be concluded that with an increase in the number of model iterations, the data transmission success rate of network models constructed using various optimization algorithms gradually improves. However, the IMCN model based on the CRO algorithm outperforms others in terms of data transmission success rate. Upon further analysis of these findings, it becomes evident that distinct optimization algorithms yield significantly varying performance outcomes in the context of network models, specifically with respect to data transmission efficacy. Notably, the models formulated through the GAN and ACAN algorithms manifest relatively modest transmission success rates in their early stages. Yet, these rates exhibit a gradual improvement as the number of model iterations increases. In stark contrast, the model established upon the framework of the IMCN algorithm demonstrates a notably high data transfer success rate right from the outset, and this exceptional performance is consistently sustained as the number of iterations escalates.

### Performance evaluation and comparison of node deployment optimization algorithms for UWSNs based on network throughput and failure probability

**Figure 9:**
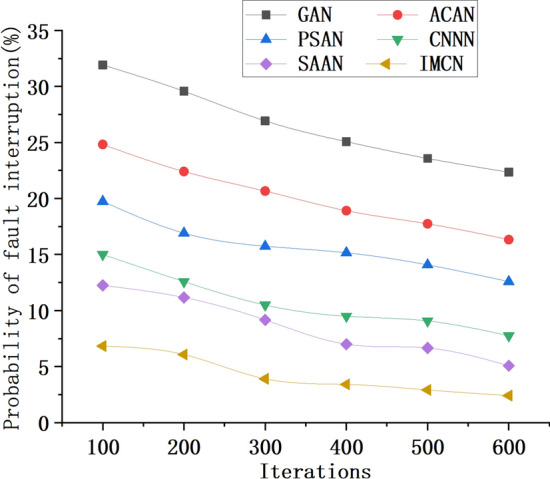
Trend of probability data change of fault interruption of UWSN model constructed by different optimization algorithms.

**Figure 10:**
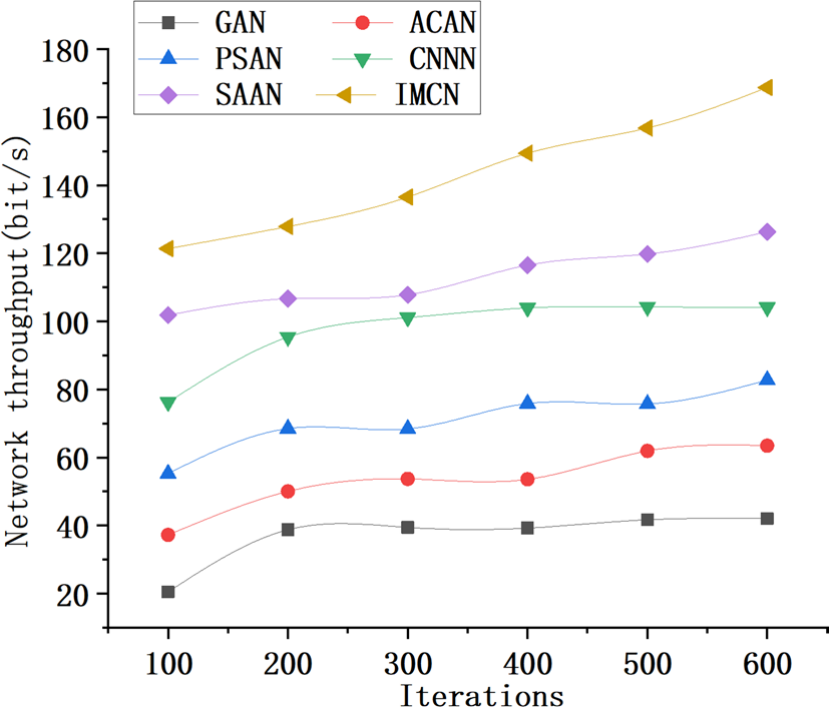
The network throughput data trend of the underwater wireless sensor model constructed by different optimization algorithms.

Figure 9 illustrates the variation in model failure probability data when constructing UWSNs using different optimization algorithms, while Fig. 10 presents the corresponding data throughput trend of the network. These figures provide valuable insights into the performance of different optimization algorithms in terms of model reliability and data transmission efficiency.

As depicted in Fig. 9, the probability of failure in UWSN models constructed using various optimization algorithms decreases notably as the number of model iterations increases. For instance, when the model iteration count reaches 600, the failure probability for the GAN-based network is 23.25%, the ACAN-based network exhibits a failure probability of 16.34%, and the IMCN-based network demonstrates a significantly lower failure probability of 2.42%. In conjunction with the experimental findings, these results confirm that the IMCN model based on the CRO algorithm outperforms other models by showcasing a superior performance with a lower probability of failure. The alteration of fault outage probability within the UWSN models fashioned by distinct algorithms becomes apparent as time elapses or other factors come into play. By juxtaposing the fault interruption probabilities within UWSN models crafted by diverse algorithms, their relative strengths and weaknesses in terms of fault handling capacity can be assessed. Thus, the IMCN algorithm emerges as efficacious in curtailing the failure likelihood within UWSN models. This algorithm conscientiously considers communication capabilities and energy limitations among nodes, utilizing an adaptive node positioning strategy that bolsters network reliability and stability. Conversely, other optimization algorithms exhibit subpar performance concerning failure probability mitigation.

As shown in Fig. 10, the network throughput of the underwater wireless sensor models constructed using different optimization algorithms exhibits an increasing trend as the number of model iterations increases. At 100 iterations, the GAN algorithm achieves a network throughput of 20.47 bit/s, the ACAN algorithm achieves 37.24 bit/s, the PSAN algorithm achieves 55.27 bit/s, the CNNN algorithm achieves 76.23 bit/s, the SAAN algorithm achieves 101.81 bit/s, and the IMCN algorithm achieves the highest network throughput of 124.47 bit/s. At 600 iterations, the IMCN algorithm reaches its peak performance with a network throughput of 174.94 bit/s, surpassing the other algorithms that exhibit limited effects. The superior performance of the IMCN algorithm can be attributed to its ability to adapt to the complex underwater environment, enhance sensor node coverage, and improve network reliability. The aforementioned results highlight the superior network performance and efficiency of the IMCN model based on the CRO algorithm. It outperforms other algorithms in terms of network throughput, enabling better utilization of available resources. These findings underscore the suitability of the IMCN algorithm for UWSNs, providing improved coverage of sensor nodes and enhancing network reliability. Figure 10 depicts the temporal evolution of network throughput in relation to the number of iterations. This visualization elucidates the varying degrees of efficacy exhibited by different algorithms in augmenting network throughput. The trajectory of throughput data within underwater wireless sensor network models, developed via distinct optimization algorithms, is influenced by multifarious factors, encompassing signal fading, the multipath phenomenon, and congestion control. In comparison to the other considered models, the proposed IMCN algorithm stands out with optimal performance, yielding a superior underwater wireless sensor network model characterized by enhanced throughput.

Furthermore, to provide a more lucid analysis of the node deployment and network optimization efficacy across diverse models, the evaluation outcomes of performance metrics for various models are systematically compiled and presented in Table 1.

**Table 1:** Impact of different optimization algorithms on UWSN performance.

Optimization algorithm	Node deployment energy consumption (J)	Node deployment time (s)	Coverage rate (%)	Successful data transmission rate (%)	Failure probability (%)	Network throughput (bit/s)
GAN	189	3.2	92.72	66.13	23.25	20.47
ACAN	145	4.8	85.67	70.44	16.34	37.24
IMCN	127	4.0	91.71	95.35	12.42	174.94
PSAN	134	3.6	86.89	89.68	16.54	161.14
CNNN	139	4.6	89.46	86.37	18.49	78.63
SAAN	126	2.9	93.12	93.67	23.12	86.83

In summary, the IMCN algorithm, utilizing the CRO algorithm, stands out in UWSN performance by demonstrating attributes of low energy consumption, swift deployment time, extensive coverage, elevated data transmission success rate, minimal failure probability, and superior network throughput. As such, the choice to employ the IMCN algorithm proves effective within specific application scenarios.

## Conclusion

The fast progress of IoT technology and intelligent optimization algorithms have created a pressing need for efficient and accurate data acquisition and transmission in underwater environment monitoring, marine resource exploration, and marine scientific research. UWSN technology, as a novel monitoring method, holds immense potential for real-time underwater environment monitoring and data collection. This study addresses the demand for adaptive and enhanced data acquisition in UWSNs by leveraging intelligent optimization algorithms and collaborative robot strategies. Specifically, an adaptive enhanced hybrid intelligent optimization algorithm combining genetic algorithm and particle swarm optimization algorithm is employed to achieve a more comprehensive and precise initial layout. Moreover, an intelligent machine collaborative node deployment model based on the CRO algorithm is proposed. The results demonstrate notable energy consumption improvements in node deployment using the proposed models. When the model reaches 600 iterations, the energy consumption for GAN-based node deployment is 189 J, while ACAN-based node deployment consumes 145 J. In contrast, the energy consumption for IMCN-based node deployment is merely 27 J. Additionally, the improved CRO algorithm yields an average coverage rate of 95.66%, surpassing other traditional intelligent optimization algorithms. This achievement substantially enhances the monitoring capability, resource utilization efficiency, and environmental monitoring accuracy of underwater networks. Nevertheless, this study does have certain limitations, particularly in the evaluation system for intelligent optimization algorithms, which could benefit from further refinement. Future investigations should expand the model dataset’s size, thereby further improving the coverage of sensor networks and the effectiveness of data transmission.

### Supplementary Information

Supplementary Information.

## Data Availability

All data generated or analysed during this study are included in this published article (and its supplementary information files). If someone wants to request the data from this study please contact the Corresponding author (Yangmei Zhang, 201707019@xaau.edu.cn).
